# Piezoelastic PVDF/TPU Nanofibrous Composite Membrane: Fabrication and Characterization

**DOI:** 10.3390/polym11101634

**Published:** 2019-10-10

**Authors:** Eman Elnabawy, Ahmed H. Hassanain, Nader Shehata, Anton Popelka, Remya Nair, Saifallah Yousef, Ishac Kandas

**Affiliations:** 1 Center of Smart Nanotechnology and Photonics (CSNP), SmartCI Research Center of Excellence, Alexandria University, Alexandria 21544, Egypt; ch.eman.elnabawy@gmail.com (E.E.); eng-saifallah.youssef1621@alexu.edu.eg (S.Y.); 2 Department of Textile Engineering, Faculty of Engineering, Alexandria University, Alexandria 21544, Egypt; ahassan@ncsu.edu; 3 Department of Engineering Mathematics and Physics, Faculty of Engineering, Alexandria University, Alexandria 21544, Egypt; r.nair@kcst.edu.kw; 4 Department of Physics, Kuwait College of Science and Technology (KCST), Jahraa 13133, Kuwait; 5 Faculty of Science, Utah State University, Logan, UT 84341, USA; 6 The Bradley Department of Electrical and Computer Engineering, Virginia Tech, Blacksburg, VA 24061, USA; 7 Center of Advanced Materials (CAM), Qatar University, Doha 2713, Qatar; anton.popelka@qu.edu.qa

**Keywords:** Piezoelectric, mechanical characteristics, nanofibers, elasticity, PVDF, polyurethane

## Abstract

Poly (vinylidene fluoride) nanofibers (PVDF NFs) have been extensively used in energy harvesting applications due to their promising piezoresponse characteristics. However, the mechanical properties of the generated fibers are still lacking. Therefore, we are presenting in this work a promising improvement in the elasticity properties of PVDF nanofibrous membrane through thermoplastic polyurethane (TPU) additives. Morphological, physical, and mechanical analyses were performed for membranes developed from different blend ratios. Then, the impact of added weight ratio of TPU on the piezoelectric response of the formed nanofibrous composite membranes was studied. The piezoelectric characteristics were studied through impulse loading testing where the electric voltage had been detected under applied mass weights. Piezoelectric characteristics were investigated further through a pressure mode test the developed nanofibrous composite membranes were found to be mechanically deformed under applied electric potential. This work introduces promising high elastic piezoelectric materials that can be used in a wide variety of applications including energy harvesting, wearable electronics, self-cleaning filters, and motion/vibration sensors.

## 1. Introduction

Alternative energy sources [1], such as synthetic energy of mechanical movements [2], thermal energies [3], and wind and water waves [4], are widely considered by many researchers as potential candidates for different economic and environmental challenges. Environmental energies have been widely introduced as alternative green sources of energy [5,6]. They can be employed to provide electrical energy for various applications due to their tendency to extract clean electrical energy from dissipating environmental energy sources [7]. The piezoelectric effect is considered a unique property that allows materials to convert mechanical energy into electrical energy and vice versa. This particular property has been strongly supported for energy harvesting applications [8,9,10,11,12,13]. The stimulation for piezoelectric materials can be supplied by human walking, rain, wind, or waves [14]. Among several piezoelectric nanofiber materials, the particular properties of poly (vinylidene fluoride) (PVDF) nanofibers such as high flexibility, high surface area, chemical inertness, and mechanical stability make it a perfect candidate for various applications including nanogenerators [15,16,17], high sensitivity sensors [18], and actuators [19].

There are several methods that can be used to fabricate the PVDF nanofibers and their nanocomposites. The most common and controlled technique is electrospinning [20,21,22,23] due to its ability to enhance the piezoelectric response of PVDF nanofiber by inducing the β-phase through polling under high electric field [24,25,26]. Electrospinning is an emerging technique to prepare polymer membranes that are composed of ultrafine fibers with micron and sub-micron diameter [27,28]. PVDF-composite nanofiber with several additives such as carbon nanotubes (CNTs), graphene, BaTiO3, and ZnO has been widely studied for enhancing the PVDF piezoelectric performance [29]. A PVDF–ZnO nanocomposite has been synthesized by the electrospinning method to be used as a nanogenerator [30]. It has been indicated that the output voltage of nanogenerator increases from 351 mV to 1.1 V when employing ZnO nanoparticles. Another study introduced the effect of CNT addition on the piezoelectric response of aligned and non-aligned PVDF nanofibers. A significant increase in the piezoelectric sensitivity up to 73.8 mV/g with applied masses down to 100 g resulted with increasing the CNTs concentration up to 0.3 wt% [31].

Among various composite nanofibrous materials, thermoplastic polyurethane has a great potential in many applications where high mechanical characteristics are required [4,32]. These include wound healing [33], filtration [34], and sensors [35]. The polar cyanide group on the thermoplastic polyurethane (TPU) backbone chain, along with its good mechanical properties, makes it a great candidate for matrix composite nanofibers [36,37,38]. PVDF/TPU porous membrane with the addition of Polyvinylpyrrolidone (PVP) as hydrophilic polymer was introduced through the phase inversion method for bovine serum albumin (BSA) retention [39]. The results showed that the addition of different PVP concentrations (0, 3, 5, and 10 wt %) affected the pores’ formation structure. It was observed that with increasing the PVP concentration, the pyriform voids were replaced by the ‘finger-like’ or macrovoids structure, which became longer and more widespread with increasing the PVP to 5 and 10 wt%. Another study used polyurethane (PU)/PVDF electrospun scaffold for wound healing applications [40]. The piezoelectric and mechanical properties of different blend ratios (1:3, 1:1, and 3:1) were evaluated to investigate the piezoelectric effect of the scaffolds on fibroblast activities. The fibroblasts cultured on the piezoelectric-excited scaffolds showed enhanced migration, adhesion, and secretion. The scaffolds that were subcutaneously implanted in Sprague Dawley (SD) rats showed a higher fibrosis level due to the piezoelectrical stimulation. The results also showed significant improvement on the mechanical properties with increasing the TPU ratio compared to neat PVDF, while the PVDF strongly affected the piezoelectric performance and in sequence reduced the mechanical strength of the composite.

A parallel bicomponent TPU/ Polyimide (PI) membrane with enhanced mechanical strength has been introduced as an electrolyte for the lithium ion battery [41]. The structure combined the high thermal stability of PI and the good mechanical strength of TPU to investigate a promising hybrid polymer electrolyte with high porosity, high electrolyte uptake (665%), and significant ionic conductivity (5.06 mS·cm^−1^) at room temperature.

In our work, we are providing a composite of poly (vinylidene fluoride) as a piezoelectric polymer and polyurethane as a thermoplastic elastomer, in order to achieve a high mechanically robust mat for piezoelectric applications (piezoelastic NFs). The effect of thermoplastic polyurethane (TPU) addition with different ratios on the morphological structure and mechanical analysis of PVDF nanofibers was investigated, while the physical and structural properties of PVDF/TPU composite nanofiber including scanning electron microscope (SEM), Fourier transforms (FT-IR), and X-ray diffraction (XRD) analysis were introduced. The piezoelectric properties of PVDF/TPU composite nanofiber were studied through both impulse and compression loading analysis and piezoforce microscopy.

## 2. Materials and Methods

### 2.1. Materials

Polyvinylidene fluoride (PVDF) (Kynar^®^, King of Prussia, PA, USA) was supplied by ARKEMA and thermoplastic polyurethane (TPU) with Polydispersity Index (PDI) of 1.83 and 107,020 g mol^−1^ molecular weight was supplied by (BASF Co., Ltd., Berlin, Germany). Known polymer concentrations were dispersed in dimethylformamide (DMF 98%, Sigma Aldrich, Taufkirchen, Germany).

### 2.2. Membrane Fabrication

PVDF polymer solution of 15 wt.% was prepared through adding 3 gm of PVDF powder into 20 mL of DMF, while 10 wt.% of TPU polymer solution was attained by dispersing 2 gm of TPU pellets into 20 mL of DMF. Different blend ratios of PVDF/TPU composite polymers were prepared (1:1, 1:3, and 3:1) through polymer blending for 24 h before the electrospinning process.

Electrospinning was performed by adding 5 mL of polymer solution into a plastic syringe tipped with a stainless steel needle. The positive voltages were provided from a high voltage power supply CZE1000R (Spellman, Hauppauge, NY, USA) to the metallic needle with gauge 18, for application of voltages around 25 kV with constant feed rate of (1 mL/h) using a NE1000 syringe pump (New Era Pump Systems, Suffolk County, NY, USA). Needle-to-collector distance was 10 cm. Random PVDF/TPU nanofibers composite was collected on a drum collector covered with aluminum foil and connected to the ground.

### 2.3. Morphological Characterization

The morphology of PVDF/TPU nanofibers (NFs) was observed by scanning electron microscope (JEOL JSM-6010LV-SEM, Tokyo, Japan) with an accelerating voltage of 15 kV. The nanofiber mats were placed on a carbon tape fixed on aluminum stubs and sputter coated with platinum. The diameter of NFs was analyzed using Image-J software (Madison, WI, USA). The average fiber diameter distribution was manually detected by measuring the length through fiber boundaries at different imaging scales (50 µm, 10 µm, and 1 µm).

### 2.4. Physical Characterization

The crystal phase of NFs was obtained with an X-ray diffractometer (XRD) (Shimadzu Xlab 6100, Kyoto, Japan) with Cu Kα (1.5 Å) radiation over Bragg angle from 10° to 90°. The β phase content was calculated with the aid of a Fourier transform infra-red spectrometer (FT-IR) (Vertex 70 FT-IR, Bruker, Billerica, MA, USA) in ATR mode. Samples were scanned 120 times at a resolution of 5 cm^−1^ over a range of 4000–400 cm^−1^.

### 2.5. Mechanical Characterization

**Figure 1:**
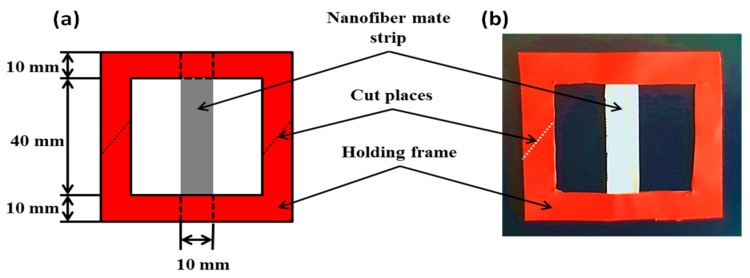
Mechanical test set-up. (**a**) Schematic diagram of the cardboard holding frame used for testing and (**b**) real view of the cardboard holding frame with test sample.

Testing the effect of TPU addition into the PVDF mechanical properties was performed by cutting the nanofiber mat into rectangular pieces (1 × 6 cm). The samples were placed between two cardboard holding frames with gauge length of 4 cm as shown in Figure 1. A universal testing machine (TENSO LAB 5000, Mesdan, Italy) was used to perform the tensile test. The tensile test was conducted at a strain rate equal to 10 mm/min with zero initial loads. The load cell used was equal to 100 N.

### 2.6. Piezoelectric Measurements

#### 2.6.1. Impulse Load Test

**Figure 2:**
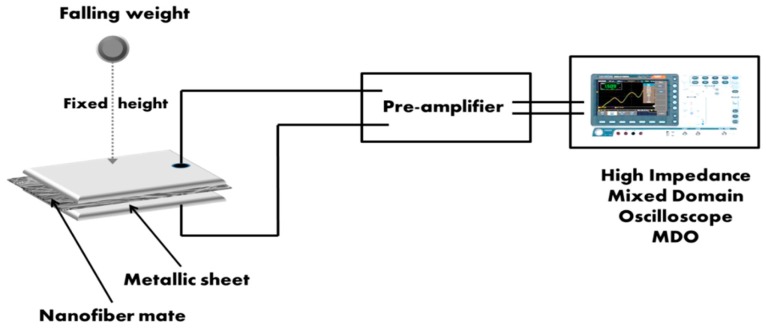
Schematic of impulse loading set-up.

Piezoelectric voltage signals were detected from the synthesized PVDF/TPU nanofibers mats through a simple set-up of an impulse loading test, as shown in Figure 2. Nanofibers mats of dimensions 2 × 2 cm were placed between two foil sheets and exposed to impulse loading test of different weights. In this testing, different weights fell down on the sandwiched mats from a fixed small height of 1 cm. Then, the generated voltage was detected through two connected shielded wires, pasted on the foil sheets, to a pre-amplifier (Stanford, CA, USA) followed by a high impedance mixed domain oscilloscope (Tektronix MDO 3012, Beaverton, OR, USA).

#### 2.6.2. Pressure Test

In this part, mechanical pressure was applied through a spring which was controlled by an electric motor that could control the pressure frequency of the applied spring in a range of a few Hz. Regarding force control, the spring compression was controlled and mapped to corresponding force values. The spring plunger had a circular light-rubber disc of 2 cm diameter. The nanofibers mat was then sandwiched between two foil sheets and the generated voltage detected through the same high impedance oscilloscope, as mentioned in Section 2.6.1.

#### 2.6.3. Piezoresponse Force Microscope (PFM)

The formed nanofiber mats of different PVDF/TPU blends ratios were analyzed using an atomic force microscopy (AFM) system MFP-3D (Asylum Research, High Wycombe, UK) with a single-frequency piezoresponse force microscope (PFM) contact mode at the Center of Advanced Materials (CAM), Qatar University, Doha, Qatar. In this characterization, the mechanical surface deformation had been measured under applied electric voltages. To excite the sample with the electric signal, a conductive tip with platinum-deposited cantilever AC240TM (Olympus, Tokyo, Japan) had been used. The tip, of 2 N/m spring constant and 70 kHz resonance frequency, was first calibrated using thermal GetRealTM mode to obtain an exact value of the spring constant and accurately convert the raw signal in (V) to picometer (pm) with applying voltage range from 1 V up to 10 V, and the subsequent surface roughness amplitude response was recorded and evaluated using Igor Pro 6.37 software (Wave Metrics, Portland, OR, USA).

## 3. Results

### 3.1. Morphological Characterization

**Figure 3:**
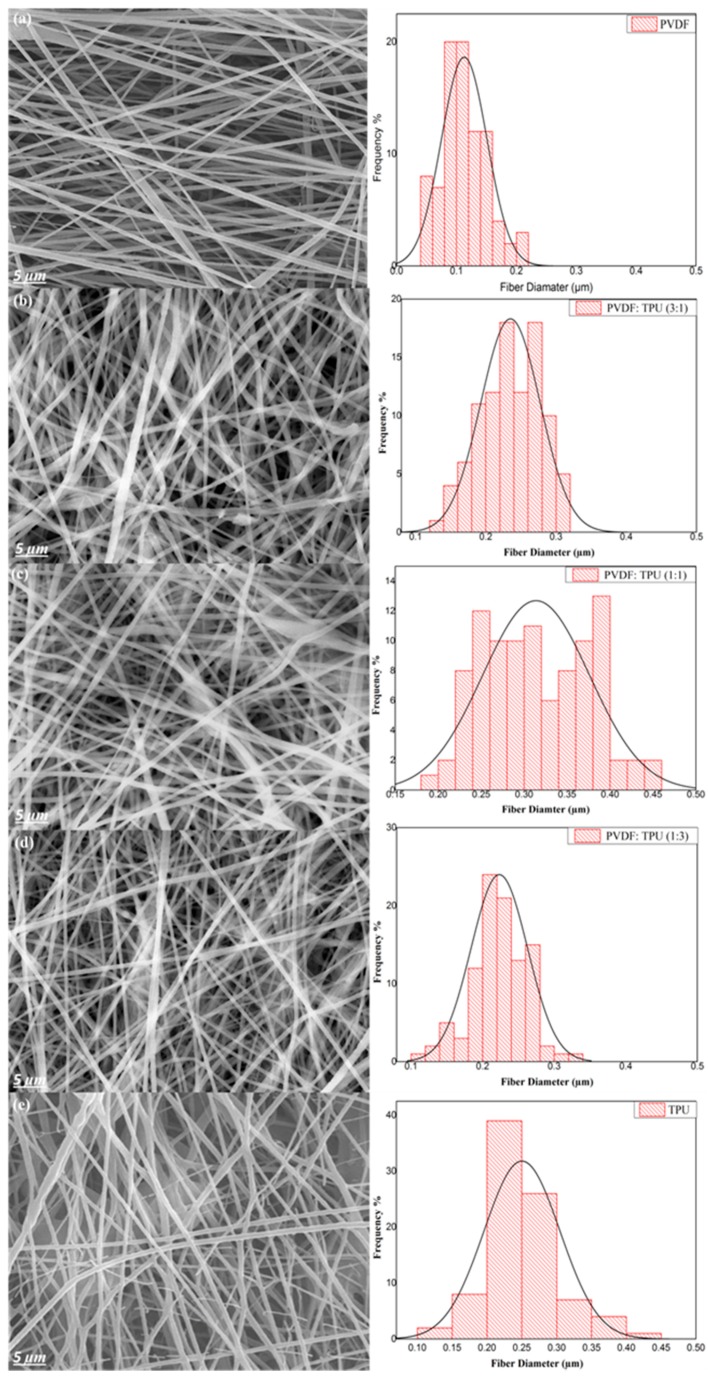
SEM images with fiber diameter distribution for PVDF/TPU composite nanofiber. (**a**) PVDF (**b**) PVDF/TPU 3:1, (**c**) PVDF/TPU 1:1, (**d**) PVDF/TPU 1:3, and (**e**) TPU.

PVDF/TPU composite nanofibers morphology was examined by Field Emission Scanning Electron Microscope (FESEM). From the respective micrographic images, quantitative analysis of fiber diameter and diameter size distribution was conducted. Average fiber diameter of nanofibers for all samples was calculated by ImageJ as represented in Table 1. The images are illustrated in Figure 3a–e, revealing the impact of TPU addition on the fiber formation and fiber morphology. As shown in Figure 3, normally distributed and beads-free fibers were obtained in all cases as a result of optimized spinning conditions and homogenous polymers blending. The spinning conditions for bead-free nanofibers fabricated from PVDF, TPU, and PVDF/TPU solutions were adjusted as follows: polymer solution feeding rate 1 mL/h, applied voltage 25 kV, needle tip to drum distance 10 cm, ambient temperature 20 ± 5 °C, and relative humidity 65%. In addition to the previous spinning conditions, polymer solution concentration had a large effect on nanofiber morphological features. In this work, polymer solution concentrations were optimized to be 15 and 10 wt.% for PVDF and TPU, respectively.

The effect of TPU addition on the morphological structure of PVDF/TPU nanofibrous composites was investigated. It was noticed that larger fiber diameter and wide diameter size distribution resulted from pure TPU and PVDF/TPU nanofibrous composites compared to the pure PVDF. The average fiber diameter of pure PVDF nanofibrous composite membrane was 110 nm while the average fiber diameters of PVDF/TPU (1:1) and pure TPU were 311 nm and 275 nm respectively.

### 3.2. Crystalline Phase Characterization

**Figure 4:**
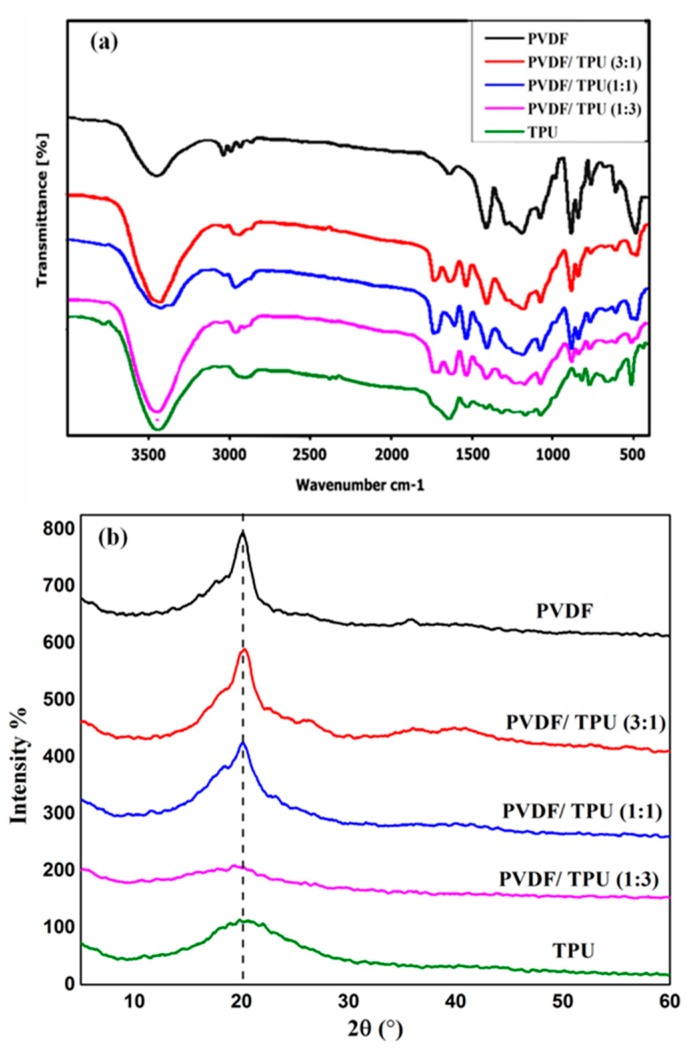
(**a**) FT-IR and (**b**) XRD Analysis of PVDF/TPU Composite Nanofiber.

The FT-IR spectra of nanofibrous composite membranes are shown in Figure 4a. The FT-IR combined with XRD can implement the identification of the crystalline phases of PVDF. PVDF can be formed in five crystalline polymorph phases (α, β, γ, δ, and ε). The α-phase is considered the most common and stable non-polar phase of PVDF while the β phase content is an essential prerequisite for the enhancement of the piezoelectric properties. Through the electrospinning process, the high electric field induces the dipoles to be aligned in the same direction normal to the chain axis. This crystal form can therefore generate the largest spontaneous polarization and exhibits a strong piezoelectric effect. Thus, the β-phase is the most important phase for piezoelectric and pyroelectric applications [6,42]. As shown in Figure 4a, the graph shows the characteristic bands for the C–F vibration of PVDF at 1191, 1400, and 881 cm^−1^. Moreover, the absorption modes for the β-phase at 840 cm^−1^ appeared strongly for PVDF nanofibers and decreased with increasing the TPU concentration, which was consistent with the decreasing of piezoelectric response with the addition of TPU. The relative amount of β phase has been quantified by considering the relative absorption intensity of β phase at 840 cm^−1^ and α phase at 760 cm^−1^ according to the proposed equation by Gregorio and Cestari [43,44]:

F (β) = Aβ/(1.3Aα + Aβ)(1)

where F (β) represents the β phase content and Aα and Aβ are the absorbance at 766 and 840 cm^−1^ respectively. By calculating the previous equation according to the obtained IR curves, the β-phase content for the PVDF nanofiber is 0.75, which confirms the high piezoelectric response of pure PVDF, while a noticeable decrease in the β-phase is observed with increasing the TPU concentration to 0.48 for PVDF:TPU (1:3) nanofiber.

The XRD graph (Figure 4b) reveals that the main characteristic peak of β PVDF appeared at 20.6° for the PVDF and PVDF/TPU composite nanofiber [40,45] whereas a slight shift occurred in the case of TPU nanofiber. The main peaks of β PVDF that resulted from the XRD and FT-IR analysis also confirmed the piezoelectric behavior of blended PVDF/TPU composite.

### 3.3. Mechanical Analysis

**Figure 5:**
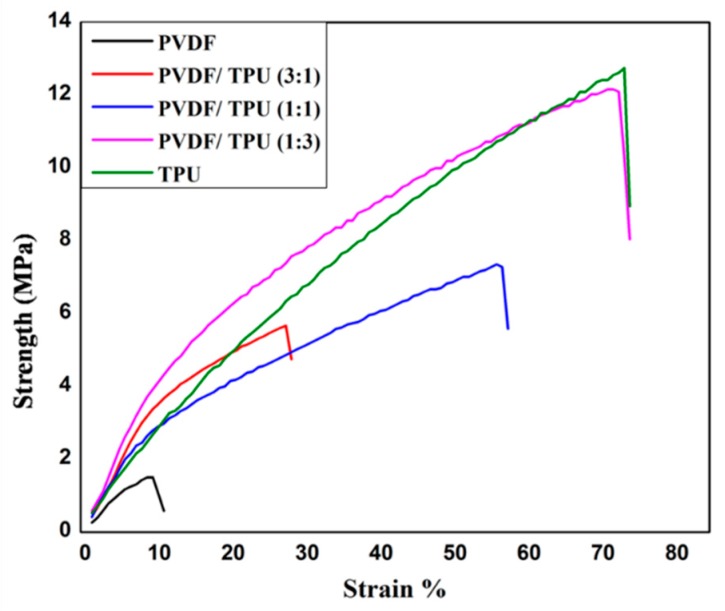
Stress strain curve of PVDF: TPU composite nanofiber.

In order to explore the mechanical properties of the newly developed composite, a tensile strain test was conducted and the recorded stress-strain curve is presented in Figure 5. It was clearly noticed that TPU and PVDF/TPU 1:3 exhibited the highest mechanical properties with maximum stress of 14.98 and 13.2 MPa respectively and breaking strain of 97.25% and 85% respectively. This can be attributed to the excellent mechanical properties of TPU as an elastomer polymer with high tensile stress and elasticity.

As it can be seen in Table 2, the maximum strength and breaking strain of PVDF are 1.63 MPa and 12.25%, while the PVDF/TPU (3:1) sample had a maximum strength of 7.49 MPa and elongation at break of 45.5%. That means adding 25% of TPU increases the strength around fivefold, while the elongation at break, which means the elasticity of the sample, increases around fourfold. By increasing the ratio of TPU to 50% as in PVDF/TPU (1:1), maximum strength increases sixfold while breaking strain increases six times compared to pure PVDF nanofibrous sample.

Toughness of nanofibrous composites membranes was calculated from the area under the stress-strain curves. As known, toughness is the ability of material to absorb energy and deform without fracture. It can be obviously noticed that by increasing the ratio of TPU, toughness is increased. This means that the developed membranes are getting more elastic and able to absorb more energy. As mentioned earlier, this can be explained by the excellent mechanical properties of TPU which is considered as one of the elastomer polymers.

### 3.4. Piezoelectric Analysis

**Figure 6:**
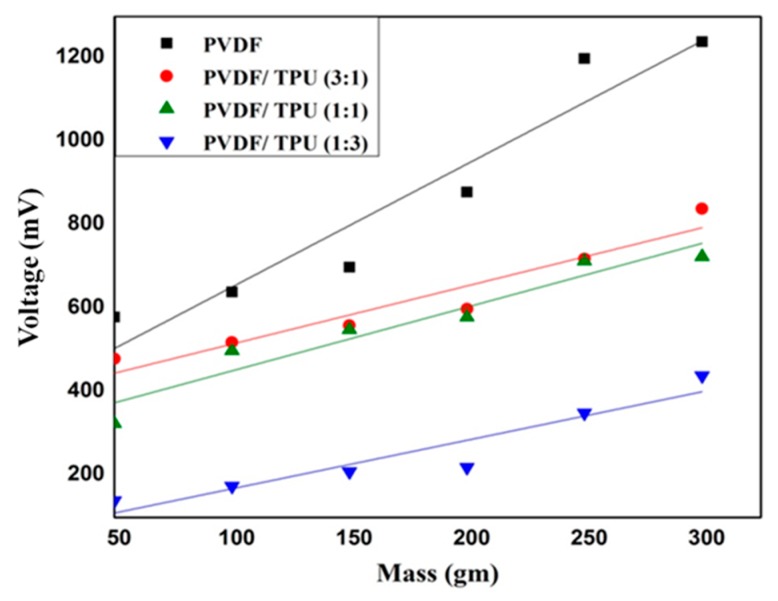
Piezoelectric response of different PVDF/TPU nanofibrous composite membranes under different impulse weight loading from 1 cm height.

**Figure 7:**
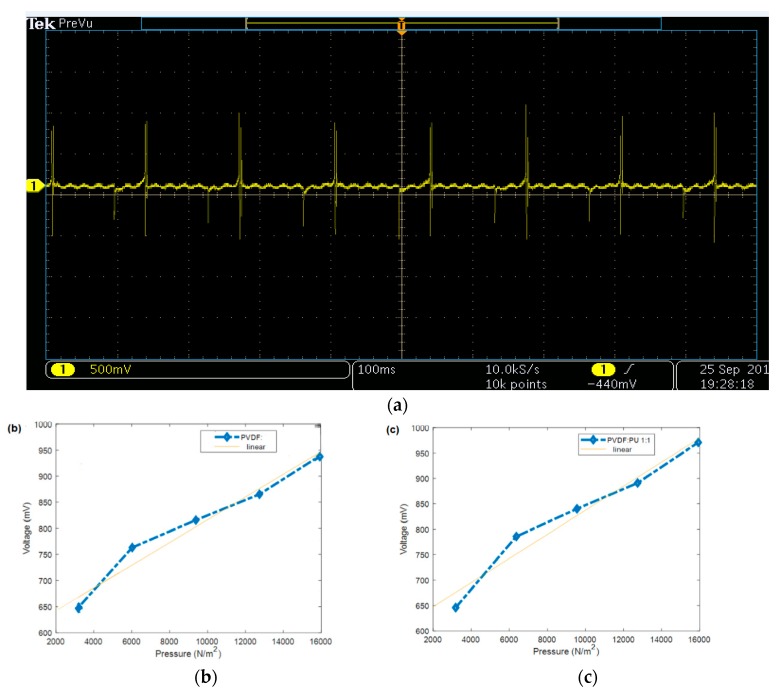
(**a**) Generated periodic voltage from one PVDF:TPU sample under periodic applied pressure, and examples of the relation between peak-to-peak output voltage and the applied pressure for (**b**) pure PVDF and (**c**) PVDF:TPU of 1:1.

Regarding the piezoelectric measurements, Figure 6 shows the piezoelectric response of different PVDF/TPU nanofibrous composite membranes under impulse loading impact from a fixed height of 1 cm. Within all samples, it could be observed that the generated electric potential response increased with increasing the exposed weight. Although the weights were thrown from a very short height (1 cm) which means very small impact force would be generated, electric potential was generated and increased by increasing the dropped weights. The pure PVDF had the highest values of generated voltage and higher sensitivity of 2.9 mV/gm compared to other samples. By increasing the ratio of TPU in the nanocomposite, the piezoelectric sensitivities were reduced as shown in Figure 6. Table 3 summarizes the impulse loading piezoresponse measurements along with the toughness results. Figure 7a shows the generated periodic voltage from one PVDF nanofiber mat under applied periodic pressure according to a force of 1 N. The mean peak-to-peak voltage was measured according to the change of applied force and consequently the applied pressure. We found that there was a close linear relation between generated voltage and applied pressure with sensitivity up to 25 mV/kPa, or corresponding to 70 mV/N, as shown, for example, in Figure 7b,c.

### 3.5. Piezoelectric Force Microscopy

**Figure 8:**
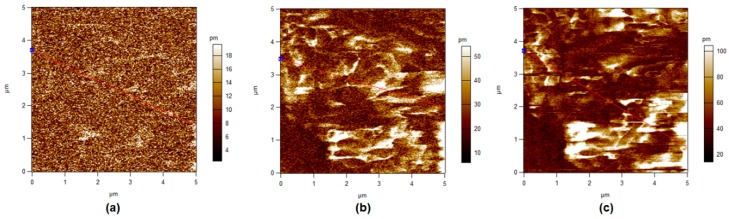
Piezoresponse force microscope (PFM) amplitude retrace of PVDF at different applied voltages. (**a**) 1 V, (**b**) 5 V, and (**c**) 10 V.

**Figure 9:**
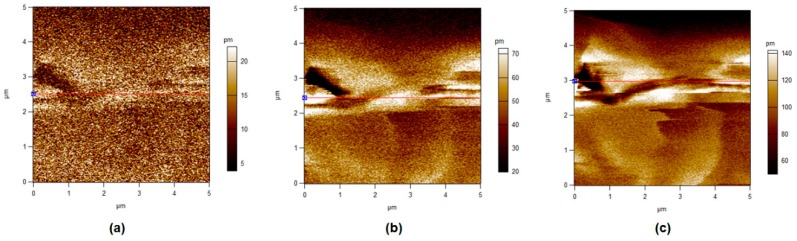
PFM Amplitude retrace of PVDF/TPU (3:1) at different applied voltages. (**a**) 1 V, (**b**) 5 V, and (**c**) 10 V.

**Figure 10:**
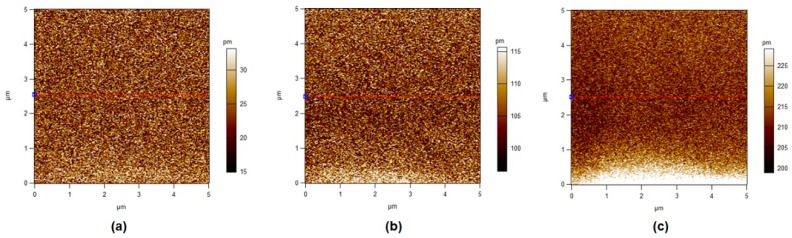
PFM Amplitude retrace of PVDF/TPU (1:1) at different applied voltages. (**a**) 1 V, (**b**) 5 V, and (**c**) 10 V.

**Figure 11:**
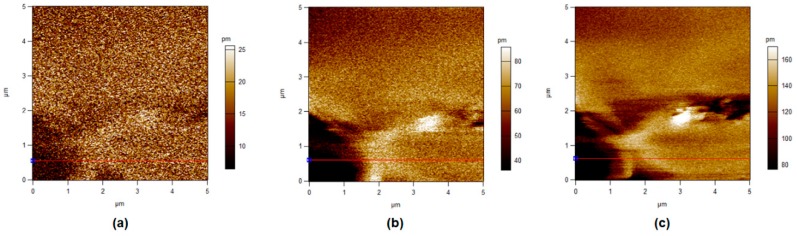
PFM Amplitude retrace of PVDF/TPU (1:3) at different applied voltages. (**a**) 1 V, (**b**) 5 V, and (**c**) 10 V.

**Figure 12:**
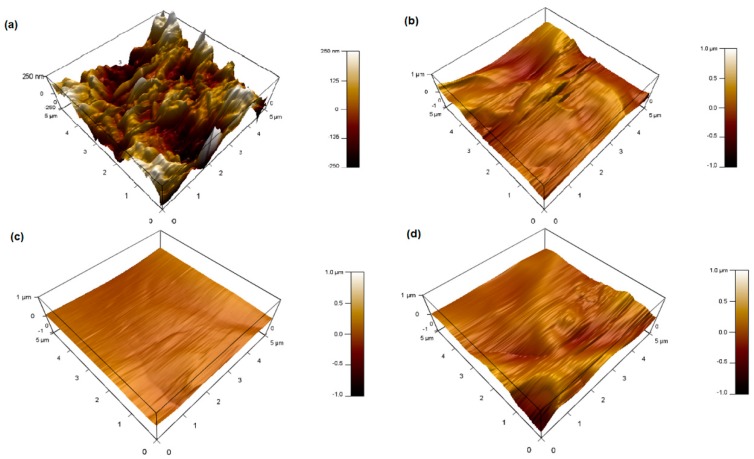
PFM Height retrace at 10 V applied potential for different PVDF/TPU blend ratios. (**a**) PVDF, (**b**) PVDF/TPU (3:1), (**c**) PVDF/TPU (1:1), and (**d**) PVDF/TPU (1:3).

In this section, different operated voltages were applied through a conductive tip to form sensitive mechanical deformations detected by PFM. Figure 8, Figure 9, Figure 10 and Figure 11 show the amplitude retrace of our synthesized different compositions of PVDF/TPU nanofibrous composite membranes at different applied voltages. Figure 12 shows the full map of surface deformation height retrace at applied 10 V for the different PVDF/TPU compositions. As a general conclusion for all samples, the formed dipoles inside the nanofibers mostly became more stretched (longer) and caused higher mechanical deformation amplitude when raising the applied electric potential. However, the addition of TPU increased in a very clear way the surface deformation under applied electric voltage, with a maximum amplitude retrace in case of PVDF/TPU (1:1), compared to all other compositions: PVDF pure, PVDF/TPU (3:1), and PVDF/TPU (1:3). Hence, the mechanical elasticity feature of the added TPU contributed to a better response of surface mechanical deformation under applied voltage. Table 4 summarizes the mean amplitude retrace for different blend ratio of PVDF/TPU nanofibrous composite membranes at different applied voltages.

## 4. Discussion

Based on SEM morphology study, the increase in diameter with increasing the TPU concentration can be attributed to the higher molecular weight of TPU polymer which leads to higher viscoelastic force of the spinning solution. Similar results are consistent with our finding that the increase in the molecular weight consequently increases the resultant fiber diameter due to a higher number of chain entanglements and increased viscosity [43]. Koski et al. also concluded that the elongation tendency of polymeric chains during electrospinning becomes more difficult with increasing the molecular weight, which decreases the splitting of the spinning jet and leads to an increase in fiber diameter [44]. Both XRD and FT-IR measurements proved that the main peaks of β-sheets of PVDF/TPU existed, which gives an indication of the availability of piezoelectric properties inside the blended nanocomposite.

Although there was enhancement of mechanical properties by increasing TPU, the generated potential and correlated sensitivity were found to be reduced which proves that the PVDF ratio with included polarizations and beta-sheets was dominant in generating electric potential under mechanical pressure excitation. Similar results have confirmed our findings by calculating the piezoelectric coefficient of PVDF/TPU blended NFs for wound healing [40]. The results showed significant decrease in d33 from 24.9 for neat PVDF to 8.26 for PVDF:TPU (1:3). Another study has introduced hybrid nanogenrator (NG) structure of PVDF, TPU, and PEDOT:PSS–PVP [46], while the flexibility of the NG was enhanced through the addition of TPU. The pyroelectric and piezoelectric properties of NG have shown that impact frequencies of 1.2 and 3 Hz could directly light a white LED. That gives an advantage to the blended composite with blend ratios of PVDF/TPU of both 1:1 and 1:3 which have reasonable piezosensitivity in addition to improved mechanical properties as shown in Table 3. In addition, our piezoresponse of PVDF:TPU membranes give a better sensitivity of up to 70 mV/N, compared to other recent PVDF:carbon nanotube piezoresponse which was limited by less than 10 mV/N [47]. It is obvious from both mechanical and piezoelectric analysis that there is a trade-off between mechanical property enhancement and piezoelectric characteristics of the developed PVDF/TPU nanofibrous composite membranes, where PVDF/TPU (1:1) nanofibrous composite membranes were found to be the optimum blend ratios which gave reasonable piezoelectric properties with good mechanical performance.

Regarding PFM analysis, it can be concluded that a little addition of TPU elastomer polymer can enhance the mechanical response (elastic response) according to applied electric potential with most of polarizability inside PVDF still kept sensitive to the applied voltage. By increasing TPU ratio over 50%, the composite loses the polarizability and piezosenstivity starts to significantly decrease. So, it can be clarified that the excellent elastic properties of TPU helped the mechanical deformation of PVDF/TPU nanofibrous composite membranes to be tremendously developed through more flexibility of the motion of electric dipoles inside the composite nanofiber under the exposure of applied electric excitation.

## 5. Conclusions

In this paper, we have presented a newly developed nanocomposite of PVDF nanofibers blended in-situ with TPU. The results showed that normally distributed and beads-free nanofibers were obtained in all cases as a result of optimized spinning conditions and homogenous polymer blending. β-phase was found to be 0.75 for the pure PVDF nanofiber which confirms the high piezoelectric response of it, while significant decrease in the β-phase was observed with increasing the TPU concentration to 0.48 for PVDF:TPU (1:3) nanofiber. The main peaks of β phase that resulted from the XRD and FT-IR analysis of different blend ratios of PVDF/TPU nanofibrous composite membranes confirms the piezoelectric behavior of PVDF/TPU composites as well. The addition of TPU improves the mechanical properties of the nanofibrous composite membrane with maximum breaking strain up to 75%. However, it reduces the piezoresponse sensitivity of PVDF nanofibrous composite membranes. Blended compositions of PVDF/TPU 1:1 and 3:1 can be considered as the optimum blend ratio and lead to a quiet trade-off between mechanical and piezoresponse characteristics. In addition, the mechanical deformations of different blend ratios of the developed nanofibrous composite membrane have been analyzed under different applied electric potentials. The blended PVDF/PU (1:1) was found to have highest mechanical surface deformation at applied 10 V, which results from more flexibility in dipole excitations inside PVDF due to the elastic content of TPU. This work is promising to develop a high elastic piezoresponse membrane that can be used in different applications such as energy harvesting, biomedical, self-cleaning filters membranes, and sensing applications.

## Figures and Tables

**Table 1:** Average Fiber Diameter of Nanofibers for Different PVDF/TPU Composite Membranes.

Sample	Poly (Vinylidene Fluoride) (PVDF)	PVDF/Thermoplastic Polyurethane (TPU) (3:1)	PVDF/TPU (1:1)	PVDF/TPU (1:3)	TPU
Average fiber diameter (nm)	110 ± 13	230 ± 25	311 ± 40	212 ± 18	275 ± 80

**Table 2:** Mechanical Properties of Nanofibrous Composites Membranes.

Sample	Max. Strength, (MPa)	Elongation at Break, (%)	Toughness, (J·m^−3^)
PVDF	1.63 ± 0.2	12.25 ± 3.6	14.2 ± 8.9
PVDF/TPU (3:1)	7.49 ± 1.8	45.5 ± 18.7	252.3 ± 152
PVDF/TPU (1:1)	8.34 ± 1.3	68 ± 12	389.3 ± 171
PVDF/TPU (1:3)	13.20 ± 3.6	85 ± 21.8	792 ± 434
TPU	14.98 ± 3.1	97.25 ± 21.3	921 ± 384

**Table 3:** Piezoelectric properties of nanofibrous composites membranes compared to mechanical properties.

Sample	Minimum Voltage at Impulse Load of 50 gm, (mV)	Maximum Voltage at Impulse Load of 300 gm, (mV)	Piezoresponse Sensitivity (mV/gm)	Toughness, (J·m^−3^)
PVDF	590	1240	2.9	14.2
PVDF/TPU (3:1)	480	830	1.6	252.3
PVDF/TPU (1:1)	350	680	1.5	389.3
PVDF/TPU (1:3)	170	400	1.1	792
TPU	0	0	0	921

**Table 4:** Mean Amplitude Retrace (in pm) for Different PVDF/TPU Nanofibrous Composite Membranes at Different Applied Voltages.

Sample	1 V	3 V	5 V	7 V	10 V
PVDF	10.6 ± 4.0	16.6 ± 5.7	24.1 ± 6.6	32.5 ± 7.6	46.0 ± 9.7
PVDF/TPU (3:1)	13.5 ± 4.5	29.0 ± 8.5	61.0 ± 22.0	85.1 + 23.0	110.0 ± 31.5
PVDF/TPU (1:1)	24.5 ± 11.0	62.5 ± 12.4	104.4 ± 9.9	142.5 ± 9.5	209.9 ± 10.0
PVDF/TPU (1:3)	16.0 ± 4.9	38.0 ± 8.1	61.0 ± 12.4	83.0 ± 17.0	140.9 ± 17.8
