# Erratum: Piezoelastic PVDF/TPU Nanofibrous Composite Membrane: Fabrication and Characterization. *Polymers* 2019, *11*(10), 1634

**DOI:** 10.3390/polym12040762

**Published:** 2020-03-31

**Authors:** Eman Elnabawy, Ahmed H. Hassanin, Nader Shehata, Anton Popelka, Remya Nair, Saifallah Yousef, Ishac Kandas

**Affiliations:** 1 Center of Smart Nanotechnology and Photonics (CSNP), SmartCI Research Center of Excellence, Alexandria University, Alexandria 21544, Egypt; ch.eman.elnabawy@gmail.com (E.E.); ahassan@ncsu.edu (A.H.H.); eng-saifallah.youssef1621@alexu.edu.eg (S.Y.); ishac@vt.edu (I.K.); 2 Department of Textile Engineering, Faculty of Engineering, Alexandria University, Alexandria 21544, Egypt; 3 Department of Engineering Mathematics and Physics, Faculty of Engineering, Alexandria University, Alexandria 21544, Egypt; r.nair@kcst.edu.kw; 4 Department of Physics, Kuwait College of Science and Technology (KCST), Jahraa 13133, State of Kuwait; 5 Faculty of Science, Utah State University, Logan, UT 84341, USA; 6 The Bradley Department of Electrical and Computer Engineering, Virginia Tech, Blacksburg, VA 24061, USA; 7 Center of Advanced Materials (CAM), Qatar University, Doha 2713, Qatar; anton.popelka@qu.edu.qa

The authors wish to make a change to the published paper [1]. Regarding the author name Ahmed H. Hassanain, the last name Hassanain should be spelled Hassanin. The authors apologize for any inconvenience caused. The change does not affect the scientific results. The manuscript will be updated, and the original will remain online on the article webpage https://www.mdpi.com/2073-4360/11/9/1474.
